# Characteristics and influencing factors of ^11^C-CFT PET imaging in patients with early and late onset Parkinson’s disease

**DOI:** 10.3389/fneur.2023.1195577

**Published:** 2023-07-06

**Authors:** Fan Kangli, Zhao Hongguang, Li Yinghua, Du Xiaoxiao, Dai Yuyin, Gao Lulu, Li Yi, Sun Zhihui, Zhang Ying

**Affiliations:** Department of Neurology, First Hospital of Jilin University, Changchun, China

**Keywords:** Parkinson’s disease, the early-onset Parkinson’s disease, the late-onset Parkinson’s disease, ^11^C-CFT PET, dopamine transporter, motor symptoms, non-motor symptoms

## Abstract

**Objective:**

This study aims to explore the difference between ^11^C-methyl-N-2β-carbomethoxy-3β-(4-fluorophenyl)-tropanel (^11^C-CFT) positron emission tomography (PET) imaging in the early-onset Parkinson’s disease (EOPD) and late-onset Parkinson’s disease (LOPD), and to analyze the correlation between ^11^C-CFT PET imaging and disease duration, Hoehn & Yahr (H&Y) stage, motor symptoms, and non-motor symptoms in patients with idiopathic Parkinson’s disease (PD), so as to explore its application value in assessing the severity of Parkinson’s disease.

**Materials and methods:**

A total of 113 patients with idiopathic PD were included in this study. The patients were divided into EOPD and LOPD groups according to the age of 60 years, of which 58 were early-onset and 55 were late-onset. All patients underwent ^11^C-CFT PET imaging and manually sketched regions of interest (ROI) to delineate the caudate nucleus, anterior putamen, and posterior putamen ROI layer-by-layer, and the corresponding values were recorded. Clinical data [age of onset, disease duration, H&Y stage, total Unified Parkinson’s Disease Rating Scale (UPDRS) score, UPDRS III score, tremor score, postural instability/gait difficulty (PIGD) score, rigidity score, bradykinesia score, and Montreal Cognitive Assessment (MoCA) score] were collected from all patients. The differences in striatal ^11^C-CFT uptake between patients with EOPD and LOPD were compared, and the correlation between striatal ^11^C-CFT uptake and the clinical data of patients with idiopathic PD was evaluated.

**Results:**

The caudate nucleus ^11^C-CFT uptake was higher in EOPD than in the LOPD group (*t* = 3.002, *p* = 0.003). ^11^C-CFT uptake in the caudate nucleus in patients with PD was negatively correlated with the age of onset, H&Y stage, disease duration, total UPDRS score, UPDRS III score, rigidity score, and bradykinesia score (*p* < 0.05). The anterior and posterior putamen ^11^C-CFT uptake was negatively correlated with H&Y stage, disease duration, total UPDRS score, UPDRS III score, PIGD score, rigidity score, and bradykinesia score (*p* < 0.05).

**Conclusion:**

^11^C-CFT PET provides an objective molecular imaging basis for the difference in disease progression rates between patients with EOPD and LOPD. Secondly, ^11^C-CFT PET can be used as an important objective indicator to assess disease severity and monitor disease progression.

## 1. Introduction

Parkinson’s disease (PD) is the second most common neurodegenerative disease worldwide after Alzheimer’s disease (AD) (1), and the main pathological changes are: degeneration and loss of nigrostriatal dopaminergic neurons, formation of Lewy bodies in the remaining neurons and reduction of striatal dopamine levels. The clinical manifestations of PD are predominantly motor symptoms, including rest tremor, rigidity, bradykinesia, and postural imbalance. With the progress of clinical and pathological research, PD has been found to be accompanied by some non-motor symptoms. Braak et al. classified the pathological changes in PD into six stages according to the different sites of α-synuclein deposition and the time and order of PD pathology in 2003, among which non-motor symptoms included: cognitive, olfactory, sleep, autonomic dysfunctions, anxiety and depression and mental and behavioral dysfunction (2). Although imaging and genetic research for the diagnosis of Parkinson’s disease have made great progress in recent years, the diagnosis of PD still depends mainly on the comprehensive assessment of medical history, symptoms and signs, and response to levodopa drugs, which lacks a gold standard for diagnosis. The pathogenesis of PD is very complex, and the pathophysiological mechanism includes: oxidative stress, mitochondrial dysfunction, neuroinflammation, etc. Based on the above pathophysiological changes, the current research hotspot is mainly to find effective biomarkers for PD diagnosis. Among them, great progress has been made in the study of biomarkers of the central nervous system and peripheral blood, and it plays an important role in the diagnosis and differential diagnosis of PD and Parkinson’s syndrome (3–5). However, it is still difficult to reflect the essential characteristics of PD. Moreover, most patients with PD have atypical clinical symptoms in the early stages, that are difficult to detect and diagnose. At the same time, a reliable objective assessment index for evaluating PD severity is lacking.

Positron emission computed tomography (PET/CT) is an *in vivo* molecular imaging technique. By combining with different radiotracers, systemic or local metabolic, functional and structural information can be obtained to achieve early detection, early diagnosis and early treatment of diseases. Different radiotracers in PET indicate different features. PET/CT is also increasingly used for the diagnosis and evaluation of PD because of its specific advantages, such as the dopamine transporter (DAT)-PET. DAT-PET imaging is based on the principle that ^11^C-methyl-N-2β-carbomethoxy-3β-(4-fluorophenyl)-tropanel (^11^C-CFT) tracers specifically bind to striatal DAT to visualize the caudate nucleus and putamen, while DAT-deficient regions such as the cerebellum and cortex do not bind to the tracer, in order to assess the density of presynaptic dopaminergic neurons in the striatum and reflect the severity of neuronal degeneration in the dense substantia nigra (6). Therefore, a reduction of ^11^C-CFT uptake in the caudate nucleus and putamen plays an important role in the early diagnosis and differential diagnosis of PD (7, 8).

DAT-PET imaging is clinically useful for differentiating PD from conditions unrelated to dopaminergic dysfunction, such as essential tremor, dystonia, drug-induced parkinsonism and vascular parkinsonism. (9, 10). However, the ^11^C-CFT uptake in different parts of the striatum in patients with PD is different, and whether it is related to the age of onset and disease severity of patients with PD, especially the correlation with motor and non-motor symptoms, needs to be further investigated. In this study, the correlation between striatal ^11^C-CFT uptake and clinical data [including age of onset, disease duration, H&Y stage, total UPDRS score, UPDRS III score, tremor score, postural instability/gait difficulty (PIGD) score, rigidity score, bradykinesia score, and the Montreal Cognitive Assessment (MoCA) score] in patients with idiopathic PD was analyzed in the Chinese population, with the aim of exploring the application value of DAT-PET in assessing PD severity. In addition, the incidence of Parkinson’s disease increases with age, and the cut-off age varies by age group, distinguished by the ages of 40, 45, 50, 60, or 70 years; the terms used in the literature, such as juvenile, young, and late-onset Parkinson’s disease, are defined differently (11). In this study, PD was defined as early-onset Parkinson’s disease (EOPD) or late-onset Parkinson’s disease (LOPD) by taking the age of onset of 60 years as the limit, and the difference between ^11^C-CFT uptake in EOPD and LOPD was preliminarily explored using DAT-PET imaging.

## 2. Materials and methods

### 2.1. Subjects

One hundred thirteen patients with a definite diagnosis of idiopathic PD who underwent ^11^C-CFT PET imaging at the Parkinson’s Disease Specialized Clinic, Department of Neurology, First Hospital of Jilin University from January 2020 to October 2022 were included in this study, including 58 patients with EOPD and 55 patients with LOPD. All patients were definitively diagnosed by an experienced Parkinson’s disease specialist according to the clinical diagnostic criteria for Parkinson’s disease established by the International Parkinson and Movement Disorders Society in 2015 (12).

Inclusion criteria were: ① clinically diagnosed idiopathic PD; ② can cooperate with ^11^C-CFT DAT-PET scan and there are no contraindications; ③ complete clinical data (including MDS-UPDRS, MoCA).

Exclusion criteria were: ① Non-idiopathic PD; ② Associated with brain diseases such as stroke, head trauma, and cranial surgery.

### 2.2. Clinical assessment

2008 MDS Revised Unified Parkinson’s Disease Rating Scale (MDS-UPDRS) was used to assess motor symptoms of PD patients (13). Among them, tremor scores included: UPDRS II 2.10, UPDRS III 3.15–3.18; rigidity scores included: UPDRS III.3.3; bradykinesia scores included: UPDRS II 2.4–2.9, UPDRS III.3.2 3.4–3.8 3.14; PIGD scores included: UPDRS II 2.12–2.13, UPDRS III.3.10–3.12 (14). Montreal Cognitive Assessment (MoCA) was used to assess cognition (15). All assessments were performed in the “on” state of the patients. The MDS-UPDRS score was performed by a professional in the patient’s “off” state, and the MoCA score was performed in the patient’s “on” state, within 1 week of the DAT PET scan.

### 2.3. ^11^C-CFT PET imaging process

The ^11^C-CFT is produced by the HM-12 cyclotron of Sumitomo Heavy Machinery Co., Ltd. in Japan with a purity of ≥95% for radiochemistry. A Siemens Discovery 16HR PET/CT scanner was used. Patients discontinued PD medications for at least 12 h before the examination, and all patients were injected intravenously with ^11^C-CFT at a uniform standard of 3.7 MBq/Kg via the back of the hand. The PET/CT examinations were performed under calm breathing after a 60-min waiting period in a quiet environment. The patient position was supine on the examination bed, the head was fixed in the head rest, and the scan area included the entire head. CT images were acquired first for attenuation correction, and PET imaging was performed in the same field of view in the three-dimensional mode for 15 min. Image reconstruction was performed using an iterative method to obtain CT, PET, and PET/CT fusion images of the brain in the transverse, coronal, and sagittal views, respectively.

### 2.4. Image analysis methods

Three consecutive images with the clearest striatal structures on the PET/CT fusion images were selected, and the regions of interest (ROI) method was used to delineate the caudate nucleus, anterior putamen and posterior putamen ROI layer-by-layer; the maximum radioactivity counts of each nucleus were recorded. The parieto-occipital cortex, which lacks DAT distribution, was selected as the background reference area, and the distribution of each ROI was semi-quantitatively calculated using the following formula: ^11^C-CFT uptake value = (ROI radioactivity count/occipital radioactivity count)-1 (16). ^11^C-CFT uptake in the caudate nucleus, anterior putamen, and posterior putamen was the average radioactivity uptake on the left and right sides.

### 2.5. Statistical analysis

SPSS26.0 software was used for the data analysis. Normally distributed data are expressed as X^- ± S, and non-normally distributed data are expressed as M (P25, P75). The *t*-test was used for comparisons between data groups that conformed to a normal distribution and the rank-sum test was used for comparisons between data groups that did not conform to a normal distribution. Analysis of covariance was used to correct the influence of confounding variables. Spearman’s correlation analysis was used to evaluate the correlation between striatal 11C-CFT uptake and the clinical data of patients with PD. *P* < 0.05 indicated a statistically significant difference.

## 3. Results

### 3.1. Subject characteristics

A total of 113 patients were included in this study, with a mean age of onset of 57.11 ± 11.53 years, and a median duration of disease of 4 (2, 6) years. They were divided into EOPD group and LOPD group according to whether the age of onset was greater than 60 years old, including 58 patients in EOPD group and 55 patients in LOPD group. There was no significant difference between groups in gender, duration of disease, H&Y stage, total UPDRS score, UPDRS III score, tremor score, PIGD score, rigidity score, and bradykinesia score. MoCA score (*p* = 0.010) and ^11^C-CFT uptake in the caudate nucleus (*p* = 0.008) were significantly higher in EOPD group than in LOPD group (Table 1).

**Table 1:** Demographics and clinical characteristics of PD patients.

Characteristic	PD (*n* = 113)	EOPD (*n* = 58)	LOPD (*n* = 55)	*P*
Participants (Male/Female)	113 (49/64)	58 (23/35)	55 (26/29)	0.165
Age of onset (year)	57.11 ± 11.53	48.41 ± 9.18	66.27 ± 4.47	<0.001
Disease duration (year)	4 (2, 6)	4 (2, 8)	3 (2, 5)	0.154
H&Y stage	2 (1, 2)	2 (1, 2)	2 (1.5, 2)	0.088
Total UPDRS score	44 (32, 67)	42.5 (31, 70)	47 (32.5, 66)	0.881
UPDRS III score	25 (18, 40)	24 (17, 40)	27 (18.5, 39)	0.419
Tremor score	7 (3, 12)	6.5 (3, 11)	9 (4.5, 13.5)	0.206
PIGD score	3 (2, 6)	3 (2, 5)	3 (2, 6)	0.582
Rigidity score	3 (2, 6)	3 (1, 7)	4 (2, 6)	0.799
Bradykinesia score	19 (12, 30)	19 (13, 31)	20 (12, 29.5)	0.847
Education (year)	11.1 ± 3.82	10.64 ± 3.44	11.58 ± 4.16	0.191
MoCA score	21.58 ± 5.06	22.86 ± 5.09	20.42 ± 4.82	0.010
Caudate nucleus	1.40 ± 0.45	1.51 ± 0.51	1.29 ± 0.35	0.008
Anterior putamen	1.20 ± 0.48	1.22 ± 0.56	1.18 ± 0.38	0.657
Posterior putamen	0.71 ± 0.46	0.73 ± 0.52	0.70 ± 0.39	0.691

### 3.2. Covariance analysis of striatal ^11^C-CFT uptake in EOPD group and LOPD group after correcting for disease duration

**Figure 1:**
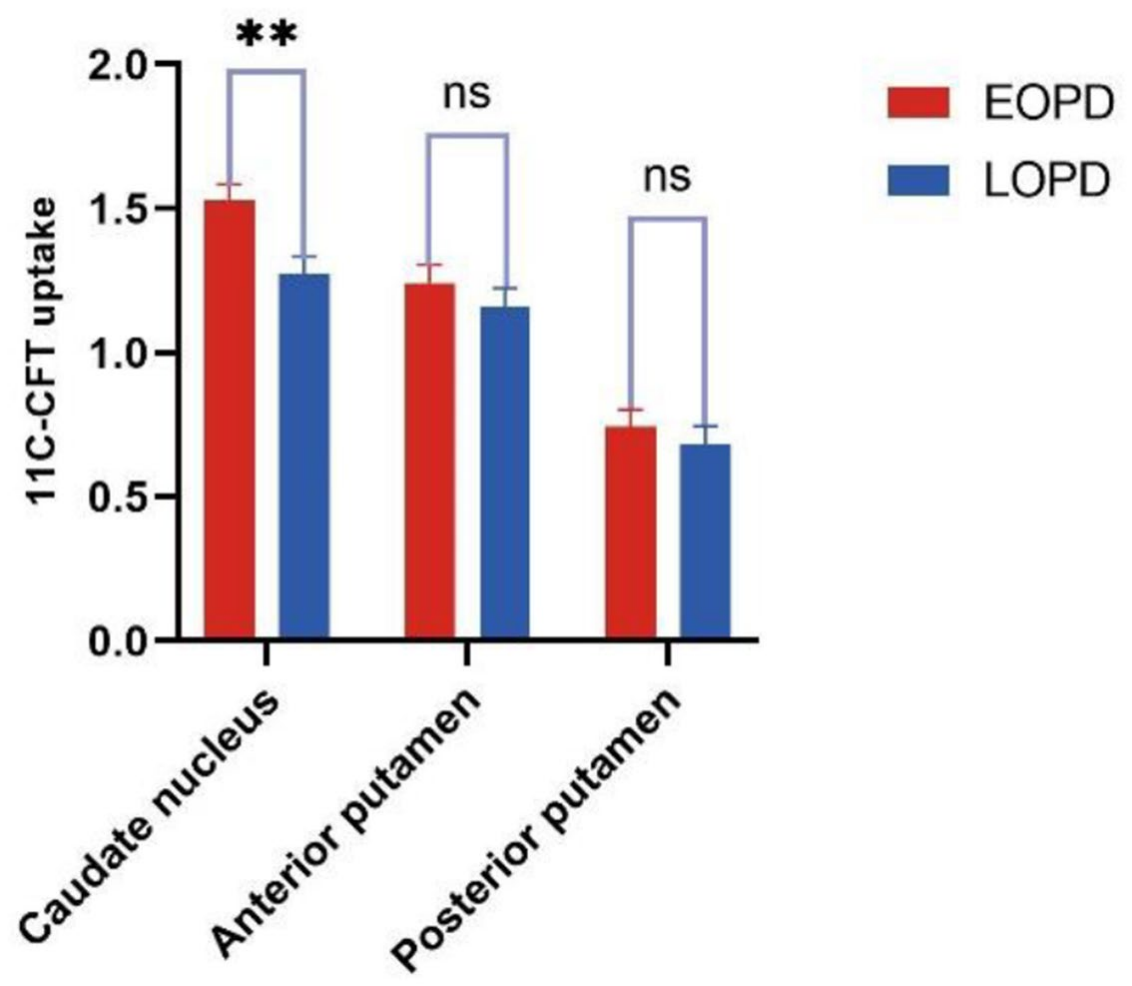
Comparation of striatal ^11^C-CFT uptake in EOPD and LOPD (**p ≤ 0.01; ns: *p* > 0.05).

**Figure 2:**
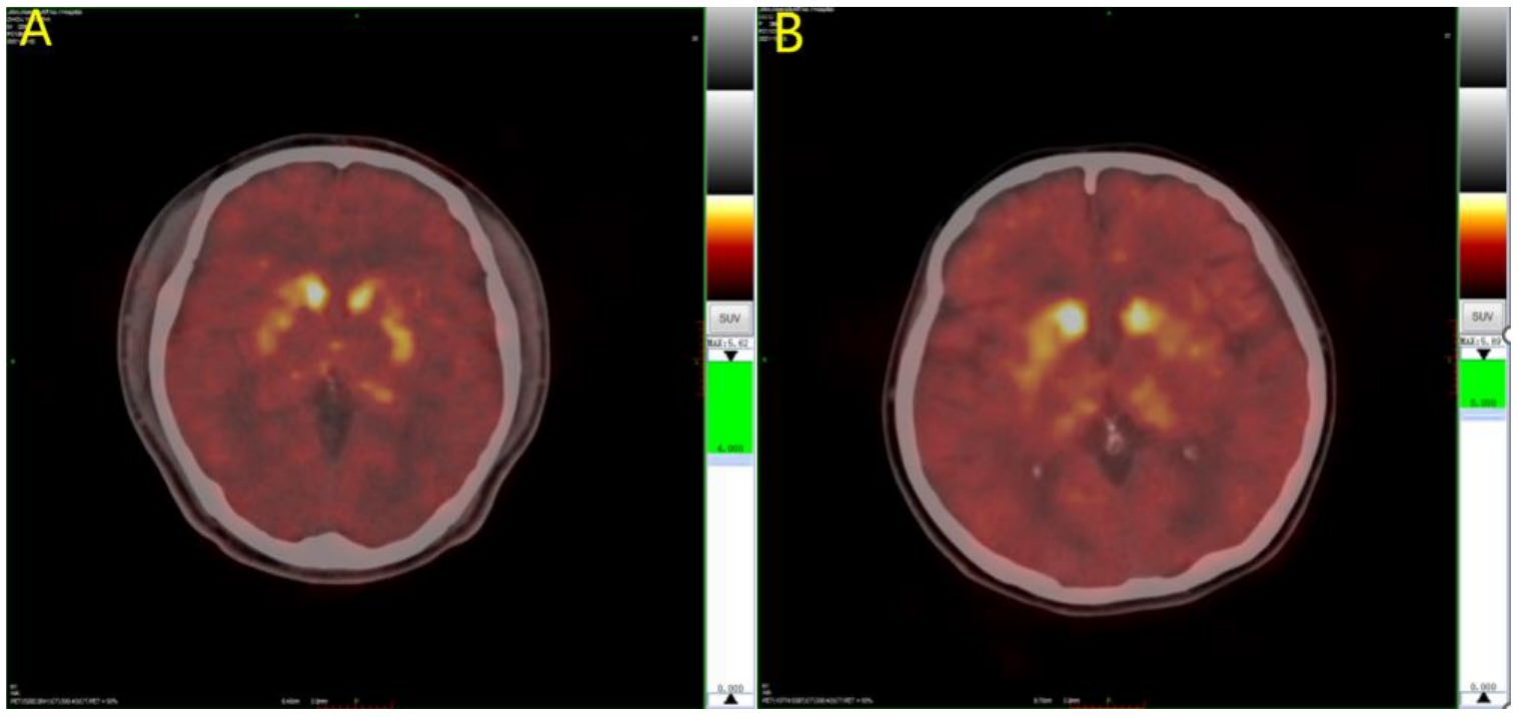
^11^C-CFT PET images of Parkinson’s disease. **(A)** LOPD; **(B)** EOPD. ^11^C-CFT PET metabolic model for EOPD and LOPD: ^11^C-CFT uptake in the caudate nucleus in patient with EOPD was significantly higher than LOPD, and there was no significant difference between the anterior and posterior putamen between patients with EOPD and LOPD.

The results of the covariance analysis after correcting for disease duration showed that ^11^C-CFT uptake in the caudate nucleus in the EOPD group was significantly higher than in the LOPD group (*t* = 3.002, *p* = 0.003). There was no significant difference between the anterior and posterior putamen ^11^C-CFT uptake between the EOPD and LOPD groups (Table 2 and Figures 1, 2).

**Table 2:** Comparation of striatal ^11^C-CFT uptake in EOPD and LOPD after correction for the disease duration.

Characteristic	Group	X ± S	*B*	*t*	*P*
Caudate nucleus	EOPD	1.527 ± 0.058	0.252	3.002	0.003
	LOPD	1.275 ± 0.059			
Anterior putamen	EOPD	1.243 ± 0.063	0.084	0.918	0.361
	LOPD	1.159 ± 0.065			
Posterior putamen	EOPD	0.742 ± 0.061	0.059	0.667	0.506
	LOPD	0.683 ± 0.063			

### 3.3. Correlation analysis of striatal ^11^C-CFT uptake in patients with PD with clinical data

**Figure 3:**
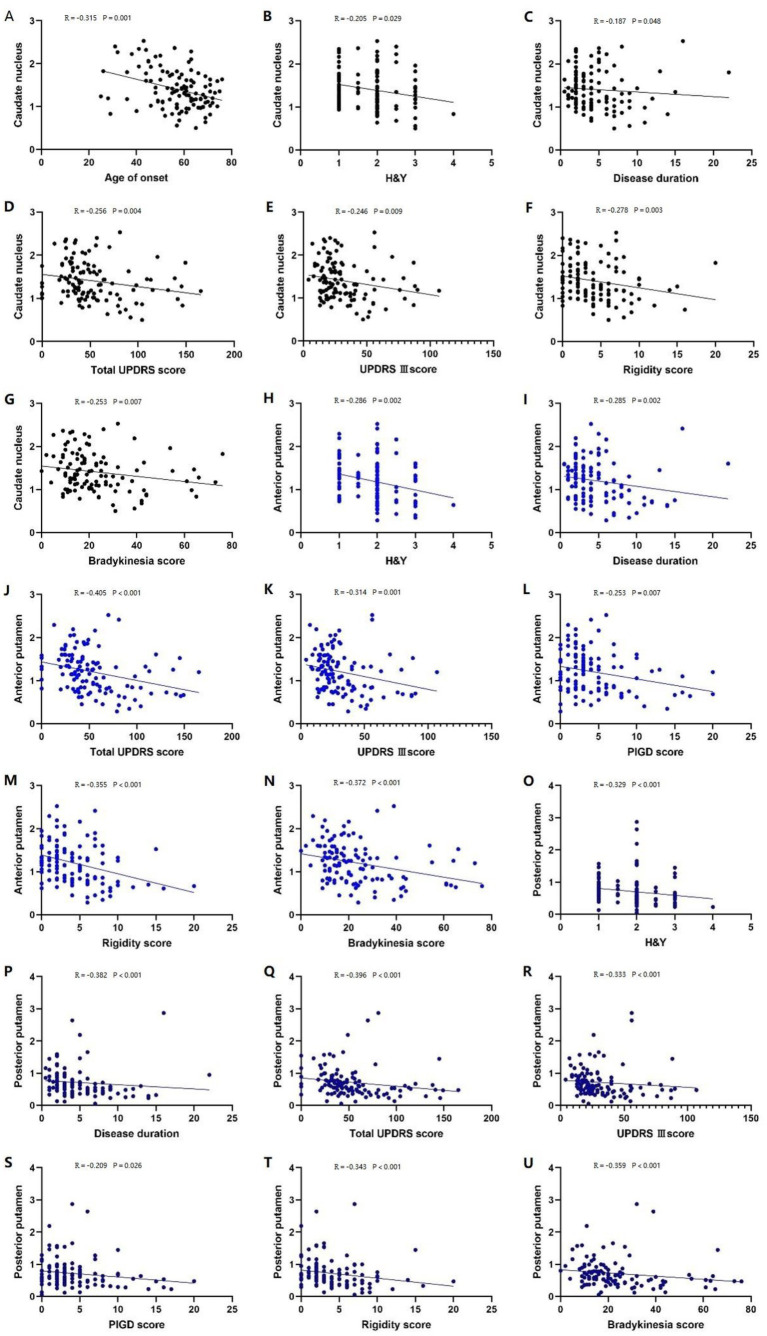
Correlation analysis of PD patients. Correlation analysis between the caudate nucleus 11C-CFT uptake and: **(A)** age of onset; **(B)** H-Y stage; **(C)** disease duration; **(D)** total UPDRS score; **(E)** UPDRS III score; **(F)** rigidity score; **(G)** bradykinesia score. Correlation analysis between the anterior putamen 11C-CFT uptake and: **(H)** H-Y stage; **(I)** disease duration; **(J)** total UPDRS score; **(K)** UPDRS III score; **(L)** PIGD score; **(M)** rigidity score; **(N)** bradykinesia score. Correlation analysis between the posterior putamen 11C-CFT uptake and: **(O)** H-Y stage; **(P)** disease duration; **(Q)** total UPDRS score; **(R)** UPDRS III score; **(S)** PIGD score; **(T)** rigidity score; **(U)** bradykinesia score.

In patients with PD, the caudate nucleus ^11^C-CFT uptake was negatively correlated with the age of onset, H&Y stage, disease duration, total UPDRS score, UPDRS III score, rigidity score, and bradykinesia score (*p* < 0.05) (Figures 3A–G), while the anterior and posterior putamen ^11^C-CFT uptake was negatively correlated with H&Y stage, disease duration, total UPDRS score, UPDRS III score, PIGD score, rigidity score, and bradykinesia score (*p* < 0.05) (Figures 3H–U). There was no significant correlation between striatal ^11^C-CFT uptake and tremor score and MoCA score (Table 3).

**Table 3:**
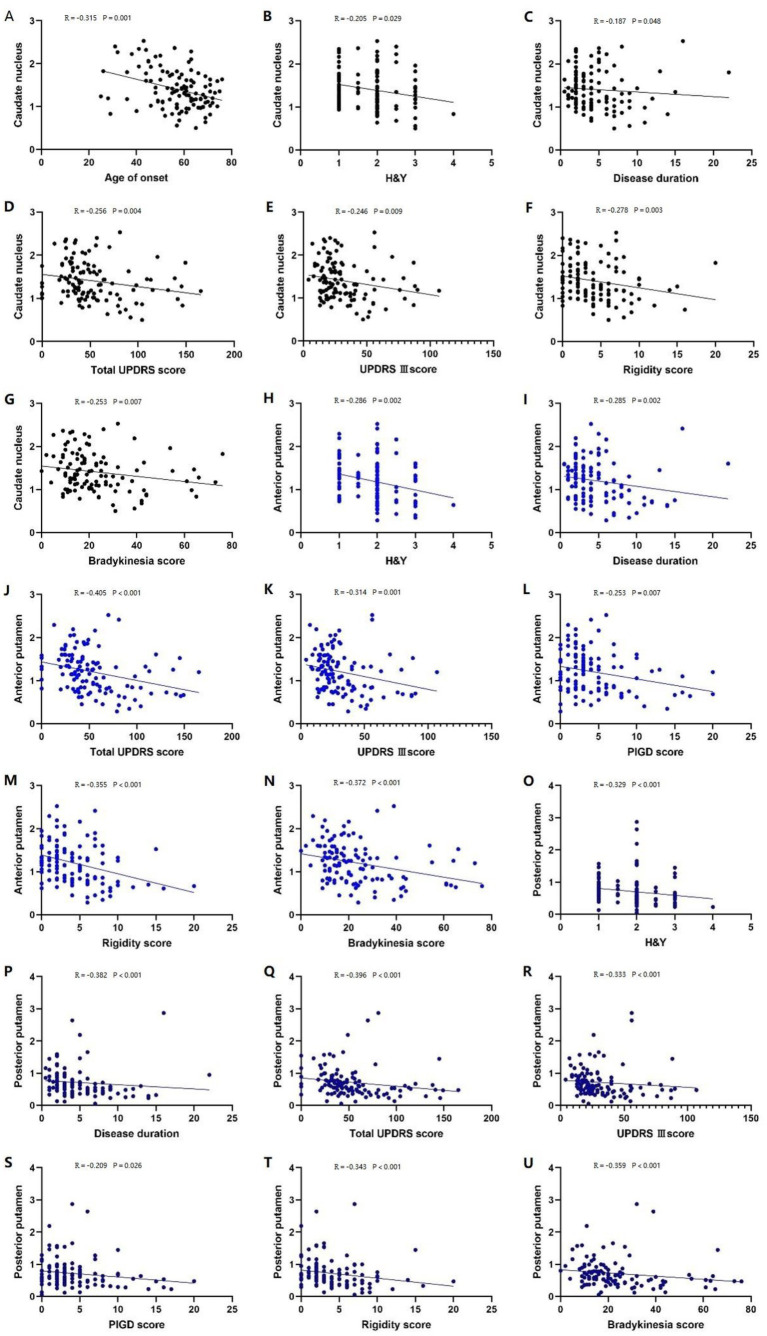
Spearman correlation analysis of striatal ^11^C-CFT uptake in PD patients with clinical data.

Characteristic	Caudate nucleus		Anterior putamen		Posterior putamen	
	*R*	*P*	*R*	*P*	*R*	*P*
Age of onset	−0.315	0.001	−0.091	0.339	−0.079	0.403
H&Y stage	−0.205	0.029	−0.286	0.002	−0.329	<0.001
Disease duration	−0.187	0.048	−0.285	0.002	−0.382	<0.001
Total UPDRS score	−0.256	0.004	−0.405	<0.001	−0.396	<0.001
UPDRS III score	−0.246	0.009	−0.314	0.001	−0.333	<0.001
Tremor score	−0.079	0.408	−0.01	0.914	−0.114	0.230
PIGD score	−0.151	0.110	−0.253	0.007	−0.209	0.026
Rigidity score	−0.278	0.003	−0.355	<0.001	−0.343	<0.001
Bradykinesia score	−0.253	0.007	−0.372	<0.001	−0.359	<0.001
MoCA score	0.065	0.495	0.068	0.477	−0.038	0.691

## 4. Discussion

PD is a chronic neurodegenerative disease characterized by the degeneration and progressive loss of dopaminergic neurons in the substantia nigra-striatum. Its prevalence increases progressively with age. DAT is located on the cell membrane of presynaptic neurons and can transport dopamine from the synaptic cleft to the presynaptic membrane for reuse or further degradation after dopaminergic neurons release impulses, thus regulating the amount of dopamine released from presynaptic nerve endings to ensure normal physiological function of the synapses. ^11^C-CFT is mainly distributed in the bilateral caudate nucleus and putamen, whereas radioactivity distribution in other regions of the brain is extremely low, indicating that dopamine in the brain is mainly concentrated in the bilateral striatal region, as consistent with the neuroanatomical dopaminergic nerve fiber projection pathway. Dopaminergic neuronal projections are mainly located in the substantia nigra-striatum pathway. Animal studies have shown that DAT significantly correlates with the levels of synaptic dopamine transmitters (17) and residual nigrostriatal dopaminergic neurons (18), which can accurately reflect the severity of nigrostriatal dopaminergic neuronal damage. The number of DAT is closely related to the occurrence and progression of PD.

First, this study found that patients with EOPD had a higher cognitive score than those with LOPD. And the ^11^C-CFT uptake in the caudate nucleus was also higher in patients with EOPD than in those with LOPD. These are consistent with the findings of Yang et al. (19). Controlling for the effects of disease duration, patients with early onset PD have higher caudate nucleus ^11^C-CFT uptake, resulting in less damage to dopaminergic neurons. It can be hypothesized that EOPD has a slower rate of disease progression, which is in agreement with Schrag et al. (20). It is also consistent with the pathological characteristics of PD, where the ventral lateral substantia nigra is most affected, dopaminergic neurons are the most deficient, and the middle and posterior parts of the putamen may receive the most projections from the ventral lateral substantia nigra; therefore, the lesion initially involves the middle and posterior parts of the putamen. As the disease progresses, the anterior putamen and caudate nucleus become progressively more involved (21). The higher ^11^C-CFT uptake in the caudate nucleus of patients with EOPD indicates less damage to dopaminergic neurons in the caudate nucleus and later involvement of the caudate nucleus, resulting in slower disease progression. Patients with EOPD sshow better cognitive function, consistent with the findings of Wickremaratchi et al. (22). Analysis of the causes, in addition to age, may be due to the unequal involvement of the caudate nucleus and putamen in parallel basal ganglia-thalamus-cortex circuits. The dopaminergic circuits associated with the putamen are primarily responsible for motor functions, and lesions in the putamen nucleus are mainly associated with motor symptoms and symptom severity (23). The cholinergic pathways associated with the caudate nucleus are more likely to be involved in cognitive and emotional functions, and patients with lesions in the caudate nucleus have cognitive changes (24). Patients with EOPD have better preservation of the caudate nucleus, and therefore, better cognition. Significant differences were observed between patients with EOPD and LOPD in terms of clinical presentation, response to medication, progression rate, and prognosis. The results of this study provide an objective molecular imaging basis for determining the differences in progression rate between the two.

Second, in this study, ^11^C-CFT uptake in the caudate nucleus, anterior putamen, and posterior putamen was negatively correlated with H&Y stage, disease duration, total UPDRS score, and UPDRS III score, consistent with previous study fundings (25, 26). In addition, this study divided the UPDRS score for movement into four subdomains: tremor, rigidity, bradykinesia, and PIGD, and found that ^11^C-CFT uptake in the caudate nucleus, anterior putamen, and posterior putamen was negatively correlated with rigidity and bradykinesia scores. ^11^C-CFT uptake in the anterior and posterior putamen negatively correlated with the PIGD score. This is related to the dysfunction of the cortex-basal ganglia-thalamus-cortex loop, in which the striatum receives fiber projections from the sensorimotor cortex and transmits them via the “direct pathway” and the “indirect pathway” (27) to the medial globus pallidus/substantia nigra pars reticulate in the output nucleus of the basal ganglia. Patients with PD have reduced substantia nigra-striatal dopaminergic, which causes inhibition of the “direct pathway” and excitation of the “indirect pathway,” resulting in motor symptoms such as reduced movement and rigidity (28). Thus, the longer the disease course, the more advanced the H&Y stage, the higher the motor symptom score, and the lower the striatal ^11^C-CFT uptake, indicating severe dopaminergic neuronal damage. Therefore, DAT PET imaging can be used to assess the severity of PD. This study also found that ^11^C-CFT uptake in the caudate nucleus, anterior putamen, and posterior putamen was not significantly correlated with tremor score, which is consistent with most previous studies suggesting that tremor is not associated with dopamine loss in the substantia nigra striatum (29–35). This may be due to the fact that the loop involved in PD tremor is not the same as the loop involved in the reduced movement and rigidity. Huang et al. showed that subthalamic burst discharges are dependent on input from the motor cortex, resulting in erroneous re-entrant information relays from the cortico-subthalamic nucleus to the pallido-thalamocortical loops, and thus Parkinsonian tremor (36). The caudate nucleus and putamen are not part of this loop, which explains why striatal ^11^C-CFT uptake does not correlate with tremor scores. In addition, pathological studies have shown that patients with tremor-type Parkinson’s disease have less degeneration in the substantia nigra, which projects mainly to the striatum, and more degeneration in the posterior region of the red nucleus, which projects mainly to the pallidus. This may also explain the lack of correlation between tremor scores and caudate-putamen DAT uptake (37).

Undeniably, ^18^F-DOPA PET is the first *in vivo* assessment of dopaminergic function in PD. ^18^F-Dopa is a fluorinated analog of levodopa that follows the same presynaptic dopamine (DA) synthesis pathway. It is decarboxylated by aromatic L-amino acid decarboxylase (AADC) and stored in presynaptic vesicles in the form of ^18^F-labeled dopamine, providing an *in vivo* measure of AADC activity and presynaptic DA storage capacity. However, in the pathological state, levodopa decreases, and AADC activity compensatively increases as a compensatory response to progressive DA cell death. Upregulated AADC activity may lead to ^18^F-dopa overestimation nerve terminal density and underestimation of the disease severity in early PD. In addition, most AADC-containing neurons are capable of taking up and converting ^18^F-dopa. ^11^C-CFT has been used to label DAT to assess dopaminergic neuron function, which may more sensitively reflect disease severity in early PD. Therefore, we chose DAT-PET in this study (38).

In conclusion, ^11^C-CFT DAT-PET provides an objective molecular imaging basis for the difference in the rate of disease progression between patients with EOPD and those with LOPD. At the same time, DAT-PET can also be used as an important objective indicator to assess disease severity and monitor disease progression. The loss of striatal DAT is closely related to the clinical manifestations, especially motor symptoms. With the help of this study, clinicians can also preliminarily estimate the extent of DAT loss and dopaminergic neuronal damage in the patient’s brain based on the patient’s medical history, symptoms, signs and scale scores to predict the severity and progression rate of the disease, which is helpful in guiding clinical treatment and prognosis. What’s more, this study provides an objective basis for screening patients for intermediate and advanced surgical indications for deep brain stimulation (DBS), and also provides an objective basis for accurate screening of patients in clinical drug trials. It is expected to promote early diagnosis and accurate treatment of Parkinson’s disease.

This study has several limitations. First, this was a single-center, small-sample study, and there may have been bias in terms of geography, race, examination, and assessment. Expansion of the sample size should be considered to conduct a multicenter study and further validate the findings. Second, DAT binding is susceptible to the effects of drugs (e.g., amantadine and modafinil) and normal aging, which may overestimate disease severity. This is intended for further validation in patients with PD who undergo DTBZ imaging.

Equations


$$\begin{array}{l} 11C\;\mathrm{CFT}\;\mathrm{uptake}\\ \;\;\;\;=\left(\mathrm{ROI}\;\mathrm{radioactivity count}/\mathrm{occipital radioactivity count}\right)-1 \end{array}$$

## Data availability statement

The original contributions presented in the study are included in the article/Supplementary material, further inquiries can be directed to the corresponding author.

## Ethics statement

The studies involving human participants were reviewed and approved by Ethics Committee of the First Hospital of Jilin University. The patients/participants provided their written informed consent to participate in this study.

## Author contributions

All authors listed have made a substantial, direct, and intellectual contribution to the work and approved it for publication.

## Funding

This work was supported by grants from the National Natural Science Foundation of China (No. 81974194) and the Natural Science Foundation of Jilin Province (No. YDZJ202201ZYTS116) to ZY.

## Conflict of interest

The authors declare that the research was conducted in the absence of any commercial or financial relationships that could be construed as a potential conflict of interest.

## Publisher’s note

All claims expressed in this article are solely those of the authors and do not necessarily represent those of their affiliated organizations, or those of the publisher, the editors and the reviewers. Any product that may be evaluated in this article, or claim that may be made by its manufacturer, is not guaranteed or endorsed by the publisher.
