# Corrigendum: Characteristics and influencing factors of ^11^C-CFT PET imaging in patients with early and late onset Parkinson's disease

**DOI:** 10.3389/fneur.2023.1289550

**Published:** 2023-09-21

**Authors:** Fan Kangli, Zhao Hongguang, Li Yinghua, Du Xiaoxiao, Dai Yuyin, Gao Lulu, Li Yi, Sun Zhihui, Zhang Ying

**Affiliations:** Department of Neurology, First Hospital of Jilin University, Changchun, China

**Keywords:** Parkinson's disease, the early-onset Parkinson's disease, the late-onset Parkinson's disease, ^11^C-CFT PET, dopamine transporter, motor symptoms, non-motor symptoms

In the published article, there was an error in the author list. Fan Kangli and Zhao Hongguang are both equal and first authors.

The authors apologize for this error and state that this does not change the scientific conclusions of the article in any way. The original article has been updated.

